# Metabolic acidosis may be as protective as hypercapnic acidosis in an ex-vivo model of severe ventilator-induced lung injury: a pilot study

**DOI:** 10.1186/1471-2253-11-8

**Published:** 2011-04-13

**Authors:** Theodoros Kapetanakis, Ilias I Siempos, Eugenios I Metaxas, Petros Kopterides, George Agrogiannis, Efstratios Patsouris, Andreas C Lazaris, Konstantinos G Stravodimos, Charis Roussos, Apostolos Armaganidis

**Affiliations:** 1 "G. P. Livanos and M. Simou" Laboratories, "Evangelismos" General Hospital, University of Athens Medical School, Athens, Greece; 2 2nd Critical Care Department, University of Athens Medical School, "Attikon" University Hospital, Athens, Greece; 3 1st Pathology Department, University of Athens Medical School, Athens, Greece; 4 1st Urology Department, University of Athens Medical School, Athens, Greece; 5 1st Department of Critical Care and Pulmonary Services, University of Athens, Medical School, "Evangelismos" General Hospital, Athens, Greece

## Abstract

**Background:**

There is mounting experimental evidence that hypercapnic acidosis protects against lung injury. However, it is unclear if acidosis *per se *rather than hypercapnia is responsible for this beneficial effect. Therefore, we sought to evaluate the effects of hypercapnic (respiratory) versus normocapnic (metabolic) acidosis in an ex vivo model of ventilator-induced lung injury (VILI).

**Methods:**

Sixty New Zealand white rabbit ventilated and perfused heart-lung preparations were used. Six study groups were evaluated. Respiratory acidosis (RA), metabolic acidosis (MA) and normocapnic-normoxic (Control - C) groups were randomized into high and low peak inspiratory pressures, respectively. Each preparation was ventilated for 1 hour according to a standardized ventilation protocol. Lung injury was evaluated by means of pulmonary edema formation (weight gain), changes in ultrafiltration coefficient, mean pulmonary artery pressure changes as well as histological alterations.

**Results:**

HPC group gained significantly greater weight than HPMA, HPRA and all three LP groups (P = 0.024), while no difference was observed between HPMA and HPRA groups regarding weight gain. Neither group differ on ultrafiltration coefficient. HPMA group experienced greater increase in the mean pulmonary artery pressure at 20 min (P = 0.0276) and 40 min (P = 0.0012) compared with all other groups. Histology scores were significantly greater in HP vs. LP groups (p < 0.001).

**Conclusions:**

In our experimental VILI model both metabolic acidosis and hypercapnic acidosis attenuated VILI-induced pulmonary edema implying a mechanism other than possible synergistic effects of acidosis with CO2 for VILI attenuation.

## Background

The ARDS Network study [1] documented that a low tidal volume (VT) of 6 ml/kg has beneficial effects on outcomes compared to the traditionally used volume of 12 ml/kg. However, these "protective" ventilation strategies may lead to the development of hypercapnic acidosis (HA), a fact initially viewed as an undesirable side effect to be corrected. Since low VT ventilation is now increasingly used in order to minimize ventilator-induced lung injury (VILI), hypercapnic acidosis, also termed "permissive hypercapnia", is frequently confronted by clinicians. Either a side-effect to be tolerated or a desirable beneficial event, hypercapnic acidosis is still an intriguing issue having received much debate.

The mounting experimental evidence for a beneficial effect of respiratory acidosis on the function of multiple organs [2] has encouraged clinicians to use low VT and peak pressure ventilation strategies in an attempt to let the carbon dioxide levels (CO_2_) rise in the arterial blood. However, it should be noted that there are no specific guidelines concerning the use of hypercapnic acidosis in clinical practice. Limited data exist concerning the outcomes of patients subjected to protective, low-volume mechanical ventilation in the context of significant acid-base status alterations. Hickling et al. [3] adopted a low tidal volume ventilation strategy and reported that the presence of acidemia (mean pH: 7.2) was related to increased survival. This suggestion was further supported by data originating from a 10-year study [4]. Kregenow et al. [5] recently reported a meta-analysis of the ARDS Network data concerning the effect of hypercapnic acidosis on survival using multivariate logistic-regression. The authors concluded that hypercapnic acidosis resulted in reduced 28-day mortality even in patients ventilated with the injurious VT of 12 ml/kg. However, this finding was not demonstrated when analysis was applied in the low VT (6 ml/kg) group.

A growing body of literature reports on diverse biological effects of hypercapnic acidosis in animal models. Shibata et al. [6] demonstrated that hypercapnic acidosis exerts protective effects to a certain extent in an isolated and perfused rabbit lung model of warm ischemia-reperfusion lung injury. In terms of capillary bed integrity, ischemia-reperfusion injured lungs were protected when hypercapnic acidosis was instituted, while HA did not alter the capillary bed status of normal, uninjured lungs. A hallmark experimental study on the effects of hypercapnic acidosis in a VILI model by Broccard et al. [7] demonstrated that it decreased the severity of VILI in an isolated rabbit lung model. Sinclair et al. [8] further supported this finding by reporting such a protective effect of hypercapnic acidosis in an in-vivo intact rabbit VILI model.

Another issue of interest is that of the role of acidosis per se (non-CO_2 _derived) versus hypercapnic acidosis in attenuation of VILI. In fact, experimental evidence suggests that the actual protective effects of hypercapnic acidosis are mediated by acidosis per se rather than hypercapnia. Laffey et al. [9] focused on the effects of hypercapnia vs acidosis per se in an ex-vivo model of warm ischemia-reperfusion rabbit lung injury. They demonstrated that both hypercapnic and normocapnic acidosis were protective in that model, but that hypercapnic acidosis exerted a more pronounced effect. Of interest, the protective effects of hypercapnic acidosis were greatly reduced if it was buffered towards a normal pH value. Moreover, Lang et al. [10] demonstrated that buffered hypercapnia, i.e. hypercapnia at normal pH, had a detrimental effect on the function of fetal rat alveolar epithelial cells when these were incubated in a cytokine-rich medium. On the other hand, the potential of acidosis *per se *to exert protective effects in various types of injury has been documented in other organ systems as well. For example, myocardial [11], brain [12] and liver [13] protection have been shown to be effectively mediated by metabolic acidosis.

Having these considerations in mind, we evaluated the effects of hypercapnic (respiratory) versus normocapnic (metabolic) acidosis in an *ex-vivo *rabbit lung model of VILI. We tested the hypothesis that metabolic acidosis is as protective as respiratory acidosis against VILI.

## Methods

### Perfusion circuit description

A previously described [14]*ex-vivo *isolated and perfused rabbit lung model of VILI was used. However, this model has been modified by our research group [15,16] in order to allow more accurate and real-time monitoring of the oxygenation and acid-base status of the perfusion fluid.

Briefly, the model consisted of a perfusion circuit incorporating the heart-lungs preparation. The perfusion medium consisted of a custom-made perfusate [15] and autologous blood. The circuit's venous pool consisted of a bottle containing the perfusion medium which was directed towards the pulmonary circulation under the drive of a mechanical pump (Masterflex 07550; Cole-Parmer Instrument Co, IL, USA). An "efferent" tube was inserted in the left atrium, and ended up over the venous pool bottle "closing" the circuit. By voluntarily adjusting the height of the end of the "efferent" tube, the afterload of the pulmonary circulation could be manipulated.

Blood pressure monitoring catheters were connected to a calibrated monitoring system (Direc/NEP 201 B Physiologic Recording System, Raytech Instruments Inc, Vancouver, BC, Canada). Additionally, real time blood gas measurement was carried out via a catheter inserted to the "afferent" tube and connected to the respective equipment (Trend Care, 7+™, TCM 7000, Diametrics Medical Inc., Minnesota, USA). A gas mixture of CO_2 _(35-45%) and room air (65-55%) was insufflated into the venous pool bottle achieving normocapnic conditions and oxygen partial pressure at the 120-170 mmHg range. Surgical extraction of the heart-lungs block and incorporation of the preparation into the perfusion circuit was performed according to a previously described technique [14]. According to the Greek legislation, approval from the Veterinary Directorate of the Prefecture of Athens in conformance to the 160/1991 Council Directive of the European Union was obtained before the beginning of the study. New Zealand white rabbits were utilized. After extraction of the heart-lungs block and through a small transverse incision at the right ventricle, an "afferent" tube was inserted into the pulmonary artery trunk and secured with sutures. Respectively, a small transverse incision was made at the apex of the heart and the "efferent" tube was advanced into the left atrium and secured with sutures and a cotton tape. Finally, the preparation was suspended from an electronic balance (BG 025, Mark-10, NY, USA) and connected to a ventilator (T-Bird AVS III, Thermo Respiratory Group, CA, USA).

### Measurements - Indices of lung injury

Lung injury was assessed using hemodynamic parameters as well as histology indices.

#### Weight gain

Weight gain represented solely the mass of the perfusate leaking through the alveolar space-blood barrier, i.e. pulmonary edema. Measurements of weight gain were made during the 1-hour period of pressure controlled ventilation (PCV) ventilation.

#### Ultrafiltration coefficient (Kf)

The integrity of the endothelium was assessed via its Kf using the double occlusion technique, previously described elsewhere [17,18].

#### Pulmonary artery pressure (PAP) changes

PAP measurements were recorded during the PCV interval, at set time points (20 and 40 min of ventilation).

#### Histology

Histology was performed on the left lung. Three tissue slices were taken from the lung apex, hilum and base. Four indices of histologic damage were evaluated similarly to other studies [8]. Intra-alveolar as well as interstitial hemorrhage, accumulation of inflammatory cells, degree of alveolar remodelling and interstitial edema were evaluated by a pathologist blinded to specimen group allocation. After evaluation of each of these parameters, a cumulative lung injury score ranging from 0 to 3 was assigned to the specimen.

### Ventilation - perfusion protocol

All preparations were initially perfused in normocapnic conditions (pCO_2 _= 40 mmHg) and maintained at a continuous positive airway pressure (CPAP) of 5 cmH_2_O. Initial flow was 30 ml/min and was progressively increased to a steady value of 300 ml/min within 20 min. A 5-min period of compensation was allowed (under CPAP = 5 cmH_2_0) and an initial measurement of the ultrafiltration coefficient - Kf (Kfbase) took place.

Within the next 30-min period under CPAP = 5 cmH_2_O, preparations were randomized to: (i) the control (C) group (pCO_2 _= 40 mmHg, pH = 7.4), (ii) the hypercapnic, respiratory acidosis (RA) group (pCO_2 _= 100-130 mmHg, pH = 6.9-7.2) or (iii) the normocapnic, metabolic acidosis (MA) group (pCO_2 _= 40 mmHg, pH = 6.9-7.2). Hypercapnic acidosis was achieved by increasing the fraction of the CO_2 _insufflated into the venous bottle from 35-45 to 60% while metabolic acidosis was achieved by slowly infusing into the perfusate medium approximately 2 ml of HCL solution 0.5 N.

Once the desirable metabolic conditions were reached and a second Kf (Kf1) measurement was obtained, the second stage of randomization took place. This included ventilation of the preparations of each metabolic group (C, RA, MA) with high pressure (HP; PCV = 22 cm H_2_O, PEEP = 3 cm H_2_O) or low pressure (LP; PCV = 12 cm H_2_O, PEEP = 3 cm H_2_O) (a total of 6 groups). All preparations were then ventilated for 1 hour. At the end of this period, a third Kf (Kf2) measurement was performed and the ventilation-perfusion was terminated. In addition, the preparation's weight was measured in order to calculate the initial lung weight. The latter was calculated by subtracting the heart, connective tissue and tracheal tube weight from the initial preparation weight, after removal of the lungs at the end of the protocol.

In order to adjust for technical failures during the delicate surgical maneuvers, heart-lungs preparations macroscopically damaged during their surgical extraction, exhibiting vascular failure during the 5-min compensation period or the first 5 minutes of PCV ventilation were excluded from the analysis. In addition, if - during the 60-min period of PCV ventilation - the heart-lungs preparations exhibited visible edema overflow from the tracheal tube due to obvious failure of the lungs' vascular beds, the ventilation-perfusion was terminated. In that case, the last recorded readings of weight and PAP were included in the analysis but Kf2 could not be performed nor change in ultrafiltration coefficient (dKf) could be calculated.

### Statistical analysis

Two-way (analysis for two independent variables and their interaction) ANOVA was used for multiple comparisons of physiologic indices of lung injury (weight gain, Kf changes as well as injury scores) for between group comparisons with least significance difference (LSD) test for within-group comparisons.

## Results

The initial protocol included 60 preparations. Of these, 2 were not included in the analysis secondary to early (within the first 5 minutes) vascular failure and 1 because of macroscopic damage in accordance to the above criteria. 22 preparations demonstrated vascular failure and their ventilation was interrupted at some point throughout the protocol. All animals as well as heart-lungs preparations were similar concerning their baseline characteristics (Table 1).

**Table 1:** Characteristics of animals and heart-lungs preparations at baseline

Variables	HPC *n = 10*	HPRA *n = 10*	HPMA *n = 10*	LPC *n = 10*	LPRA *n = 10*	LPMA *n = 10*	*P *value	
Animal weight, kg	3.3 ± 1.0	3.2 ± 1.0	3,0 ± 0.9	3,2 ± 0.8	3,2 ± 0.8	3.2 ± 0.8	0.42	
Lung weight, g	19.6 ± 1.6	20.6 ± 1.2	21.4 ± 1.2	22.0 ± 1.2	20.4 ± 1.0	21.3 ± 1.4	0.79	
Ischemic time , min	37.2 ± 2.3	34.0 ± 2.6	29.7 ± 1.4	33.2 ± 1.5	32.2 ± 2.4	30.4 ± 1.0	0.11	
pH	7.42 ± 0.02	7.42 ± 0.01	7.43 ± 0.02	7.43 ± 0.01	7.41 ± 0.02	7.58 ± 0.15	0.56	
pCO_2_, mmHg	35.9 ± 4.2	37.2 ± 2.0	37.3 ± 3.5	38.1 ± 2.2	39.1 ± 1.7	36.7 ± 1.4	0.47	
pO_2_, mmHg	175.3 ± 15.2	159.4 ± 7.4	168.1 ± 16.4	157.3 ± 14.5	218.0 ± 30.3	156.3 ± 10.5	0.11	
T, °C	36.2 ± 0.1	36.4 ± 0.1	36.1 ± 0.1	36.3 ± 0.1	36.3 ± 0.1	36.3 ± 0.1	0.77	
Mean pulmonary artery pressure, mmHg	16.7 ± 0.9	15.2 ± 1.8	18.3 ± 1.3	16.7 ± 2,3	14.0 ± 1.90	19.6 ± 1.2	0.02	
Ultrafiltration coefficient, Kfbase, g/min/mm Hg/100 g	0.36 ± 0.18	0.19 ± 0.06	0.24 ± 0.05	0.60 ± 0.39	0.25 ± 0.04	0.20 ± 0.03	0.54	

All values expressed as mean ± SEM.

No statistically significant differences were observed

HPC: high-pressure control (normocapnic); HPRA: high-pressure respiratory acidosis (hypercapnic); HPMA: high-pressure metabolic acidosis; LPC: low-pressure control (normocapnic); LPRA: low-pressure respiratory acidosis (hypercapnic); LPMA: low-pressure metabolic acidosis

### Weight gain

**Figure 1:**
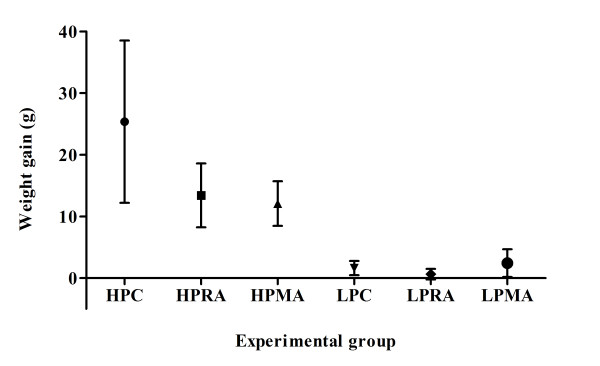
**Effects of metabolic acidosis on VILI attenuation in the HP ventilation group, in terms of weight gain i.e. pulmonary edema formation**. HPC group preparations gained significantly greater weight than their acidotic counterparts; however, both metabolic and hypercapnic acidosis had the same protective effect on weight gain

Both inspiratory pressure (P < 0.001) and pH status (P = 0.025) as well as their interaction (P = 0.024) significantly influenced weight gain. Specifically, the HPC group gained significantly greater weight than the HPMA, the HPRA and all LP groups. No difference was observed between the HPMA and HPRA groups regarding weight gain (figure 1).

### Ultrafiltration coefficient changes

**Figure 2:**
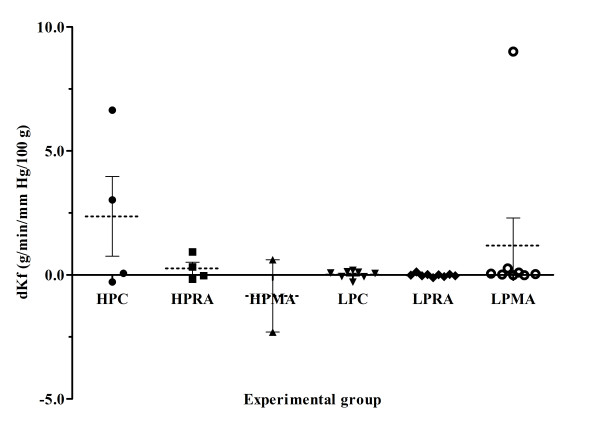
**Effects of metabolic acidosis on VILI attenuation in the HP ventilation group in terms of PAP changes**. After 20 and 40 min the HPMA group demonstrated significantly increased dPAP than its RA and C counterparts

In neither group did the dKf seem to be affected by pH (P = 0.383), pressure (P = 0.791) or their interaction (P = 0.087) (figure 2). These data are derived from 35 animals (3 for HPC, 4 for HPRA, 2 for HPMA, 8 for LPC, 9 for LPRA and 7 for LPMA group) since 22 demonstrated vascular failure, as stated above. It is true that dKf was lower with both for hypercapnic and metabolic acidosis groups, but that these differences were not statistically significant between the two.

### Pulmonary artery pressure changes

After 20 min of ventilation, the HPMA group demonstrated significantly increased dPAP than HPRA and HPC groups (*P *= 0.0276). The same applied after 40 min of ventilation (*P *= 0.0012) (Table 2).

**Table 2:** Changes in mean pulmonary artery pressure at different time points during the ventilation protocol and histology scores.

Variables	HPC *n = 9*	HPRA *n = 10*	HPMA *n = 9*	LPC *n = 10*	LPRA *n = 9*	LPMA *n = 10*
dPAP20, mmHg	8.5 ± 2.3	3,9 ± 1,9	16.7 ± 3.1	0.7 ± 1.0	0.2 ± 0.5	2.7 ± 1.7
dPAP40, mmHg	11.4 ± 2.7	10,8 ± 3.5	35.4 ± 9.9	3.2 ± 1.0	0.8 ± 0.5	5.1 ± 2.1

All values are expressed as mean ± SEM

*n *= preparations included in the study

HPC: high-pressure control (normocapnic); HPRA: high-pressure respiratory acidosis (hypercapnic); HPMA: high-pressure metabolic acidosis; LPC: low-pressure control (normocapnic); LPRA: low-pressure respiratory acidosis (hypercapnic); LPMA: low-pressure metabolic acidosis; dKf: difference in ultrafiltration coefficient before and after the 1-hr ventilation-perfusion protocol; dPAP20: difference in mean pulmonary artery pressure after 20 min of ventilation; dPAP40: difference in mean pulmonary artery pressure after 40 min of ventilation-perfusion

Repeated measures ANOVA revealed that HPMA group demonstrated significantly increased dPAP vs the HPC group (p = 0.003) while there was no difference compared with the HPRA group (p = 0.952).

### Histology

**Figure 3:**
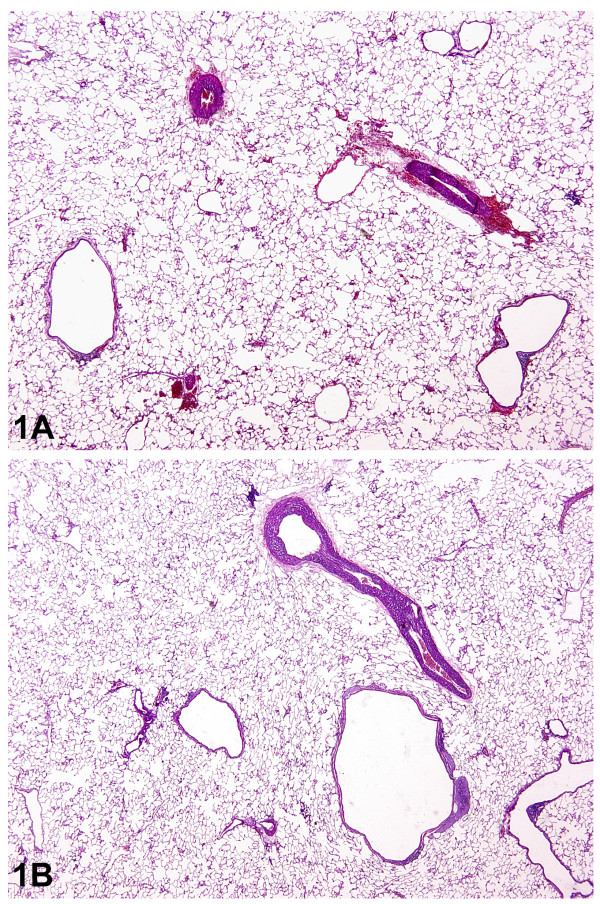
**Effects of HP (1A) vs LP (1B) ventilation on lung parenchyma-magnificationx20**. Disruption of vascular spaces and interstitial edema are more prominent in the HP ventilation group confirming the occurrence of VILI in this group

**Figure 4:**
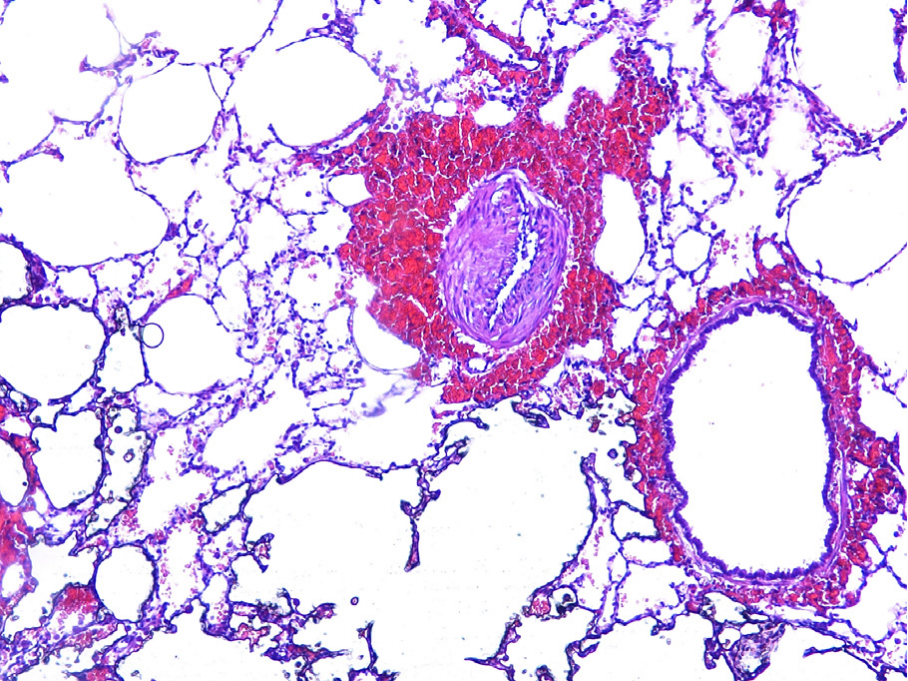
**Effects of HP ventilation on macrophage accumulation and intralveolar hemorrhage (magnificationx400)**. These effects were much mor prominent on the HP ventilation group indicating its noxious effects on normal alveolar anatomy

**Figure 5:**
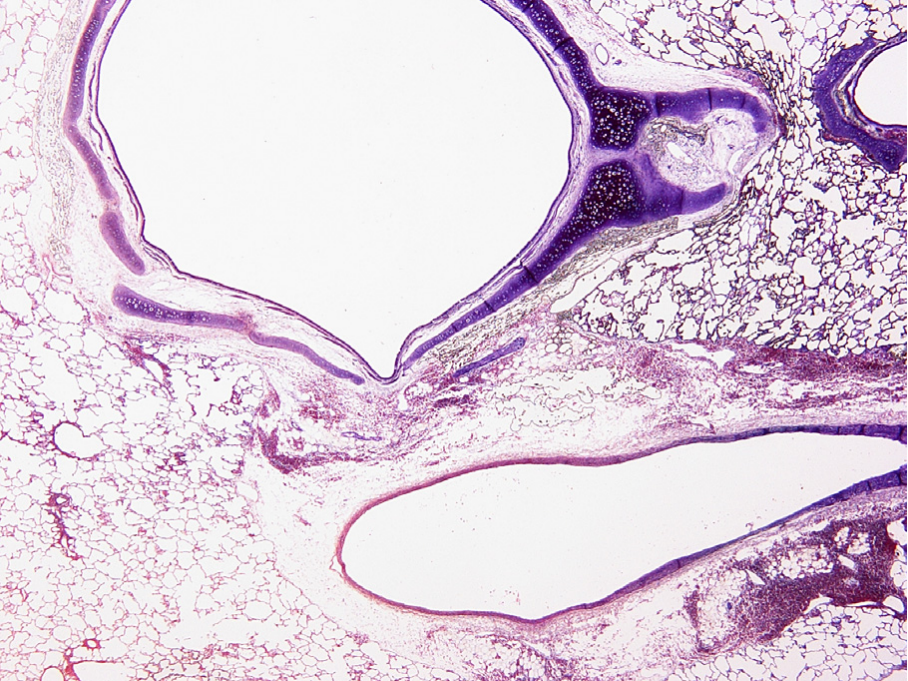
**Localization of interstitial edema in the HP ventilation group**. Large bronchi and hilar vessels were the main areas of edema formation, a phenomenon consistent in the HP ventilation group

Hemorrhage was the most prominent finding in the HP groups implying the development of VILI in HP groups (Figure 3). Macrophages were significantly accumulated only when hemorrhage was a major finding (Figure 4). Interstitial edema was a significant finding, more obvious around main bronchi and hilar vessels (Figure 5). Analysis of the lung injury scores demonstrated significantly greater injury scores for HP vs LP groups (*P <*0.001). However, pH did not seem to influence injury scores neither for HP nor for LP groups (*P *= 0.164). The latter also applied for their interaction (*P *= 0.846) (Table 2).

## Discussion

The major finding of our work was that normocapnic-metabolic acidosis was as effective as hypercapnic-respiratory acidosis in the prevention of pulmonary edema formation, a major manifestation of ventilator-induced lung injury. This pilot study implies that acidosis *per se *- either metabolic or respiratory - exerts a protective effect on experimental VILI in the isolated ventilated-perfused rabbit lung model.

The application of "protective", low volume mechanical ventilation often leads to an increase of arterial CO_2 _levels which - if not corrected by the physician - is called permissive hypercapnia and has been shown to affect multiple biological system targets. As far as lung injury is concerned, hypercapnia has effect on surfactant production [19], vascular permeability modulation [20] and NO regulation [21]; in this way, it is able to modify the severity of VILI. Overall, the current concept concerning the behavior of ventilator-injured lungs in the context of hypercapnia is that the latter exerts a protective role at least at some degree, leading to attenuated blood-gas barrier damage and therefore less pulmonary edema formation.

Broccard *et al *suggested the protective role of hypercapnic acidosis on ventilator-injured isolated rabbit lung preparations [7], a finding latter extended to the intact rabbit lung model [8]. However, to date hypercapnia has been mostly studied in relation to the concomitant acidosis it causes and - to our knowledge - few data exist concerning the effects of metabolic acidosis on VILI.

Laffey *et al *evaluated the effects of metabolic acidosis vs hypercapnic acidosis on an isolated *ex vivo *rabbit lung model of warm ischemia-reperfusion injury [9]. The investigators concluded that even though both hypercapnic and metabolic acidosis decrease the severity of ischemia-reperfusion injury, hypercapnic acidosis is more effective in doing so, a fact probably attributed to the rapid intracellular distribution of carbon dioxide versus hydrogen ions. Nomura *et al *concluded that hypercapnic, but not metabolic, acidosis improved functional recovery after cold cardioplegic ischemia in neonatal lamb hearts [22]. These data suggest acidosis as an important protective factor for various organs, mostly when it is CO_2_-derived, underlining the possible synergistic effects of CO_2 _and H^+ ^ions.

On the other hand, the work by Laffey *et al *[9] is probably the single study presenting data on the differential effects of hypercapnic and metabolic acidosis on lung tissue, yielding the need for further investigation. The latter experimental series is different from our study in several points. First, the type of lung injury is different (warm ischemia-reperfusion versus VILI). Second, the used tidal volumes were significantly different between the two studies. We used rather excessive VT (approximately 30 ml/kg) and pressures (peak 25 cmH_2_O) to achieve VILI (versus a VT of 4 ml/kg). Third, perfusate flow through the lung's capillary bed was greater in our study (300 vs 150 ml/min). Increased vascular flow is suggested to promote lung edema in the context of VILI [17]. Taking into account these considerations, it seems that we employed more injurious patterns of both ventilation and perfusion apart from elaborating on a different type of lung injury. Additionally, potential adverse effects of CO_2 _on certain settings (e.g. its contraindication in cases of increased intracranial pressure or concerns for retardation of normal vascularization and abnormal retinal neovascularization) [23] do exist. It is therefore justified to further elucidate the role of various types of acidosis on VILI and the potential for their therapeutic use in this clinical setting.

Based on previous hypotheses, one would expect hypercapnic acidosis to be more protective than metabolic acidosis. However, this was not the case in our series at least in terms of edema formation. A possible explanation for these finding could be the effect of acidosis on pulmonary vasculature. As opposed to the systemic circulation, pulmonary vasculature seems to be resistant to the vasodilator effects of acidosis [24]. Brimioulle *et al *[25] demonstrated that metabolic acidosis aggravated hypoxia-induced pulmonary artery vasoconstriction, a finding not evident with respiratory acidosis. Sada *et al *[26] reported on the vasoconstrictor effects of lactic acid-induced acidosis on the pulmonary artery of anesthetized cats. Further, acidosis seems to offset vasodilator effects of CO_2 _in the pulmonary vasculature [24]; a net vasoconstrictor effect is probably further accentuated when CO_2 _is absent. This consideration is partially supported by our results, where the high pressure-metabolic acidosis group demonstrated significantly higher increase of the mean pulmonary artery pressure than the high pressure-respiratory acidosis and control groups after 20 and 40 min of ventilation. Interestingly, the high pressure-respiratory acidosis group demonstrated consistently small increases of the mean pulmonary artery pressure; this confirms the counteracting effects of CO_2 _and acidosis on pulmonary vasculature, as suggested by other studies. By preventing the dilatation of pulmonary vasculature, thereby inhibiting abundant increase in hydrostatic drive/blood pooling in pulmonary capillaries, acidosis could probably represent a determinant of VILI attenuation.

### Project limitations and clinical significance of the experimental model

The isolated-perfused rabbit lung model used offers the ability to study the effect of several factors on lung parenchyma without the confounding effects of other organs, such as the kidney. However, this experimental strategy carries certain limitations concerning the ability to extrapolate results into clinical practice, already described in detail elsewhere [7]. Of note, the extra-thoracic location of the lungs aggravates the hyper-expansion of lung preparation probably generating even worse concomitant adverse outcomes-which is probably clinically irrelevant.

One could note that our study did not reveal the important effect of respiratory acidosis on capillary permeability, as indicated by dKf. It seems to be a contrast between the reduced weight gain and the absence of effect on dKf between HPMA/HPRA and HPC groups. A reasonable explanation is that a limited number of HP preparations actually tolerated the 1-hr period of ventilation-perfusion and, therefore, were eligible for the measurement of Kf2 and dKf; thus, data on dKf were limited and, presumably, lacked power to reveal such a difference (type b error). In Figure 2, we presented detailed data on dKf to show that HPC group exhibited a larger increase (an order of magnitude) in its Kf, the measure of the ultrafiltration coefficient, compared with both the HP-acidosis groups (respiratory and metabolic). This change paralleled the weight gain difference between these groups that was observed in a larger number of preparations.

It would be interesting to note that in regard to the effects of hypercapnic acidosis, the severity of injury might be important. The beneficial effects of hypercapnic acidosis are most pronounced in severe experimental VILI [6,7] while they are not so impressive on more clinically relevant models [27]. In addition, they appear to be absent in the setting of mild experimental VILI [28] or atelectasis [29]. Whether this is the case for metabolic acidosis is unknown, it is reasonable however to speculate that in light of these data a rather more severe model would be required to demonstrate the protective effects of metabolic acidosis.

It is now well documented that inflammatory changes occur in the lung subjected to injurious ventilation modes [30,31]. These changes are mediated by pro-inflammatory cytokines, detected both in the circulation and lung. Since this was a pilot study, elaborating on the possible physiologic effects of acidosis per se in VILI, inflammatory load was not initially addressed. However, in light of our results the latter constitutes a challenging future orientation.

## Conclusion

Our study findings suggest that metabolic, normocapnic acidosis is as protective as hypercapnic acidosis, at least in terms of pulmonary edema formation, in an *ex-vivo *rabbit lung model of VILI. This may imply that acidosis and carbon dioxide levels may exert independent effects on the attenuation of ventilator-induced lung injury.

## Authors' contributions

TK, IS, PK, EM and AA designed the investigation, reviewed the literature and drafted the manuscript. TK, IS, EM and PK conducted the experiments. TK, IS, PK, EM and AA contributed to the interpretation of the data. GA, AL and EP conducted pathology studies and interpretation.

All authors read and approved the manuscript.

## Pre-publication history

The pre-publication history for this paper can be accessed here:

http://www.biomedcentral.com/1471-2253/11/8/prepub
